# Tissue Iron Distribution Assessed by MRI in Patients with Iron Loading Anemias

**DOI:** 10.1371/journal.pone.0139220

**Published:** 2015-09-25

**Authors:** Lucía Gutiérrez, Michael J. House, Nisha Vasavda, Emma Drašar, Isabel Gonzalez-Gascon y Marin, Austin G. Kulasekararaj, Tim G. St Pierre, Swee L. Thein

**Affiliations:** 1 Instituto de Ciencia de Materiales de Madrid, ICMM-CSIC, Cantoblanco, Madrid, Spain; 2 School of Physics, The University of Western Australia, Crawley, WA, Australia; 3 King’s College London, Faculty of Life Sciences & Medicine, Molecular Haematology, London, United Kingdom; 4 King’s College Hospital NHS Foundation Trust, Department of Haematology, London, United Kingdom; 5 Hospital Infanta Leonor, Department of Haematology, Madrid, Spain; 6 NHLB/ NIH, Sickle Cell Branch, Bethesda, MD 20892, United States of America; University of Naples Federico II, ITALY

## Abstract

Bone marrow, spleen, liver and kidney proton transverse relaxation rates (R2), together with cardiac R2* from patients with sickle cell disease (SCD), paroxysmal nocturnal hemoglobinuria (PNH) and non-transfusion dependent thalassemia (NTDT) have been compared with a control group. Increased liver and bone marrow R2 values for the three groups of patients in comparison with the controls have been found. SCD and PNH patients also present an increased spleen R2 in comparison with the controls. The simultaneous measurement of R2 values for several tissue types by magnetic resonance imaging (MRI) has allowed the identification of iron distribution patterns in diseases associated with iron imbalance. Preferential liver iron loading is found in the highly transfused SCD patients, while the low transfused ones present a preferential iron loading of the spleen. Similar to the highly transfused SCD group, PNH patients preferentially accumulate iron in the liver. A reduced spleen iron accumulation in comparison with the liver and bone marrow loading has been found in NTDT patients, presumably related to the differential increased intestinal iron absorption. The correlation between serum ferritin and tissue R2 is moderate to good for the liver, spleen and bone marrow in SCD and PNH patients. However, serum ferritin does not correlate with NTDT liver R2, spleen R2 or heart R2*. As opposed to serum ferritin measurements, tissue R2 values are a more direct measurement of each tissue’s iron loading. This kind of determination will allow a better understanding of the different patterns of tissue iron biodistribution in diseases predisposed to tissue iron accumulation.

## Introduction

Anemia and ineffective erythropoiesis with consequent increased gastrointestinal absorption of iron, and frequent blood transfusions are the predominant causes of iron accumulation in patients with red blood cell disorders [1, 2]. The body lacks mechanisms for increasing excretion of the accumulated iron [3], leading to iron overload, most of which is stored in the liver. But iron may also accumulate in other organs such as the spleen, kidneys or the bone marrow [4]. The pattern of iron accumulation within the different organs appears to depend on the disease [4]. In particular, pathogenic iron species (e.g. non-transferrin bound iron (NTBI)) may appear when the plasma iron concentration exceeds the binding capacity of transferrin. NTBI is the main source of iron that generates myocardial iron overload and reactive oxygen species [5]. Although cardiac iron accumulation is frequent in transfusion-dependent β-thalassemia (TDT) patients, this effect is very unusual in sickle cell disease [6] or non-transfusion dependent thalassemia patients. The relationship between the different iron-containing species present in blood and the specific tissue iron accumulation is still poorly understood. Iron can exit some cells via the iron exporter ferroportin [7], hence iron accumulated in tissues may not remain there indefinitely. Furthermore, efficiency of iron removed in different organs varies with the different chelators used to reduce the iron accumulated in the tissues in patients with iron overload [8]. As yet, little is known about the pathways of iron flow between the different organs.

Conventionally serum ferritin measurements have been used to estimate body iron accumulation. Although this measurement can be repeated frequently, it is known that serum ferritin does not always correlate with liver iron concentration [9–11]. In addition, serum ferritin does not provide information about the relative iron accumulation in different organs [12]. A more accurate approach is a tissue biopsy [13], but this invasive procedure has associated risks [14] and cannot be repeated frequently. Magnetic resonance imaging (MRI) has been used to analyze iron accumulation in different tissues [6, 15–18]. This non-invasive technique can provide information on the concentration of iron in several tissues simultaneously. MRI methods are also well suited for longitudinal studies on iron biodistribution in which repeated measurements are needed.

In this study, we investigated the pattern of iron accumulation in liver, spleen, heart, kidneys and bone marrow in patients with sickle cell disease (SCD), paroxysmal nocturnal hemoglobinuria (PNH) and β-thalassemia intermedia (also referred to as non-transfusion dependent thalassemia, NTDT) by MRI. For this purpose, mean proton transverse relaxation rates (R2) of liver, spleen, kidney and bone marrow, and cardiac R2* have been measured as surrogate determinates of the iron concentration in the various tissues. These data have been compared with serum ferritin measurements. Iron estimated from bone marrow aspirates using Perl’s stain have also been compared with the quantitative MRI measurements in a subset of patients with PNH.

## Methods

### Study design and participants

Magnetic resonance imaging data from patients that had already had an assessment of hepatic iron loading as part of their clinical care programme and/or as part of another study approved by the NHS Research Ethics Committee (REC 05/Q0703/21), were retrospectively analyzed. The King’s College Hospital Research Ethics Committee confirmed that informed consent was not required from patients as this was a retrospective review of existing image data. Images were anonymized and de-identified prior to analysis.

Image data were available for 15 PNH patients (7 females and 8 males, aged 45.5 ± 15.7 years), all chelation naïve at the scan date. Being retrospective, there were some limitations on the analysis of the imaging data; images of the kidneys could be observed in only 3 of the 15, and cardiac R2* values were available from 14 of the 15 patients.

Image data were available for 40 chelation naïve SCD patients (25 females and 15 males, and 36 HbSS, 2 HbSβ^0^ and 2 HbSC). The average age at the date of scan was 40.2 ± 20 years. Kidney R2 data from the patients with SCD have been previously analyzed [16]. Cardiac R2* was measured for 12 of these patients. Cardiac MRI data were acquired during the same visit as the liver, bone marrow, kidney, and spleen MRI data.

Image data were available for 9 NTDT (β-thalassemia intermedia) patients (6 females and 3 males). Two of the patients had repeated scans; the time difference between the scans was 1 and 3.6 years and both measurements were considered in the study. The average age at the date of the scan was 37.8 ± 11.3 years. Cardiac R2* values, recorded during the same visit as the R2 imaging, were available for 4 of the 11 patients.

Image data for 17 healthy control participants (4 females and 13 males, aged 37 ± 7.7 years) that had already participated in another study were acquired with approval from the Fremantle Hospital Human Research Ethics Committee (08/404) and The University of Western Australia Human Research Ethics Committee to provide a reference range of normal R2 values. Being retrospective, no cardiac R2* data were available for the control group.

### Magnetic Resonance Imaging Data Acquisition and Analysis

Axial images of the abdomen covering the liver, spleen, kidneys and part of the thoracic and lumbar vertebrae were obtained from clinical MRI scanners operating at 1.5 T. Images were acquired using a single spin-echo sequence (FerriScan^®^) with 5 echo times (TE of 6, 9, 12, 15, and 18 ms), a repetition time of 2500 ms and slice thickness of 5 mm.

Spin density projection assisted R2-MRI (FerriScan^®^) [19, 20] had been used to assess liver iron concentration (LIC) in the participants. Liver R2 values were obtained from FerriScan^®^ reports. R2 values derived from pixel-wise mono-exponential fits to the image data were obtained from the FerriScan® raw image data for the bone marrow, kidney and spleen. Bone marrow values are reported as the average R2 value obtained from the vertebral body of 6 slices, corresponding in most of the patients to lower thoracic vertebrae and higher lumbar vertebrae. Kidney R2 values were determined as previously described as the average from multiple slices from both kidneys [16]. Spleen R2 values are reported as the average value from at least three different slices in each patient. While homogenous spleen signals were found in control subjects, PNH and the NTDT patients, only 24 of the 40 SCD patients showed homogeneous splenic R2 values, the lack of homogeneity most likely caused by splenic infarction. Very low standard deviations were obtained in the analysis of the different slices from each organ to obtain the mean R2 value.

Cardiac R2* data were acquired on 1.5 T MRI scanners using a breath hold gradient echo sequence with 8 echo times between 2.97 ms and 21.68 ms, a repetition time of 200 ms and slice thickness of 10 mm. R2* values were derived from pixel-wise bi-exponential fits to the image data after subtraction of background noise in quadrature.

### Clinical Data

Clinical data were collected retrospectively from the Electronic Patient Records (EPR) system and clinical notes. Seven PNH patients had bone marrow aspirate performed as part of the routine work-up; iron stores were scored (anonymized by AGK) after Perls’ staining following standard protocols [21, 22].

### Statistical Analysis

Statistical analysis was performed by GraphPad Prism Software (CA, USA). Normal distributions of R2 and R2* values for each organ and group of patients were checked using Kolmogorov-Smirnov test. Several data sets (Control BMR2, SCD Liver R2, SCD Spleen R2, PNH BMR2, SCD Ferritin, PNH Ferritin and NTDT Ferritin) did not pass this test and therefore significant differences were checked using Kruskal Wallis analysis with Dunn post test for the comparison of all the tissues R2, R2* and R2 ratios. Correlations between tissue R2 in different organs and between R2 values and serum ferritin measurements were assessed using Pearson’s test for normally distributed variables and Spearman’s test for non normal distributions. The threshold for significance was P = 0.05 and P values < 0.05 (*), < 0.01 (**) and < 0.001 (***) were considered as significant.

### Limitations

One limitation of this retrospective study is that patient selection bias is very likely. Patients were selected because they had an assessment of hepatic iron loading by MRI as part of their clinical care programme. Thus the studied cohort will not necessarily represent the complete spectrum of PNH, SCD or NTDT patients and, in particular, those patients who were not referred for a liver iron MRI measurement. A future prospective study could address this issue.

MRI analysis to quantify liver and cardiac iron accumulation is a technique validated by the U.S. Food and Drug Administration (FDA). This technique has not been validated against tissue biopsies for the analysis of other organs yet. Spleen, kidney and bone marrow R2 values are measured as surrogate values of the iron concentration.

## Results

### Comparison of tissue R2 values between patients and the control group

**Fig 1:**
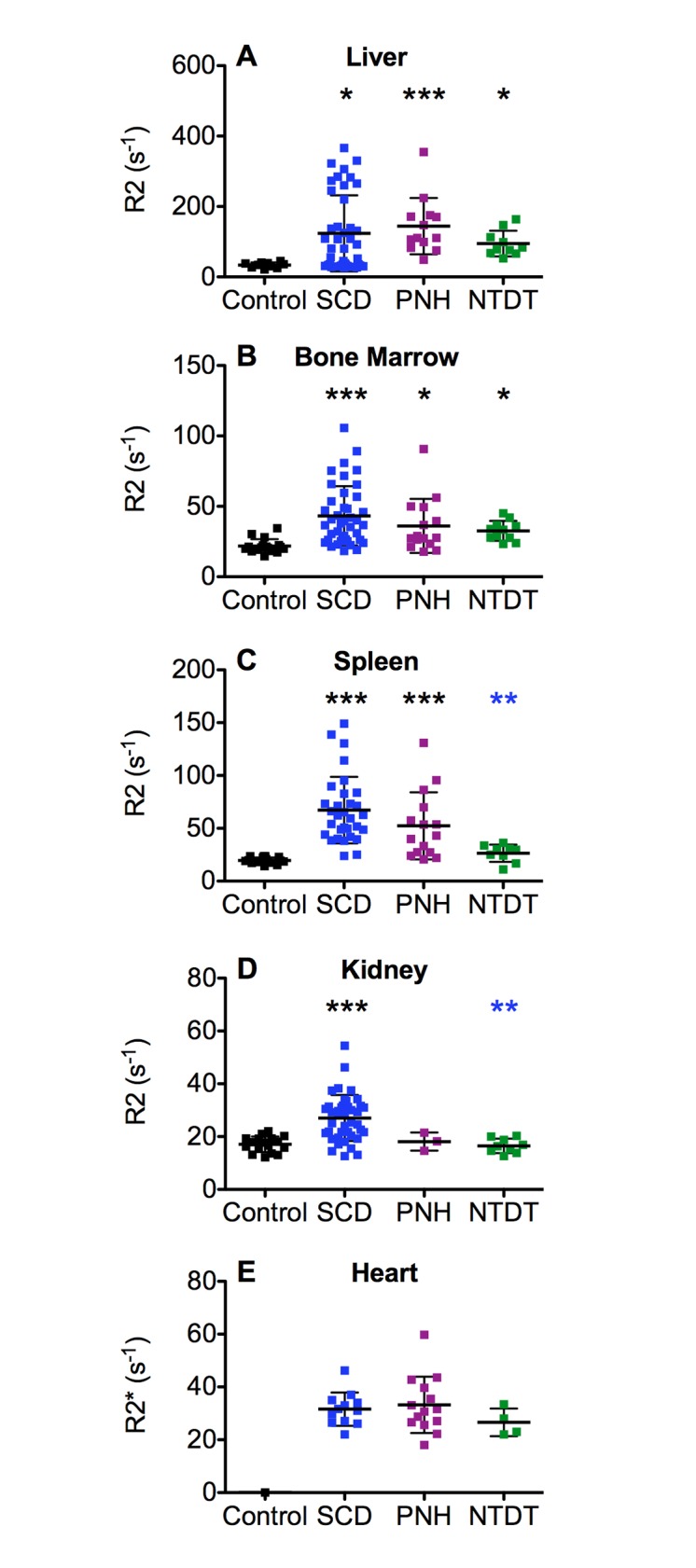
Proton transverse relaxation rates (R2) for (A) liver, (B) bone marrow, (C) spleen, (D) kidneys and (E) heart for the four different groups of subjects: control subjects (black), SCD (blue), PNH (purple) and NTDT (green) patients. Mean values ± SD are represented by the horizontal bars.(*) p < 0.05, (**) p < 0.01, (***) p < 0.001 for Kruskal Wallis analysis with Dunn post test. The asterisk color indicates the group with which the difference was found.

The mean liver and bone marrow R2 values in controls were significantly lower from those in patients (Fig 1 and Table 1). The mean R2 values for the liver were similar between the three groups of patients (Fig 1A) as were the mean bone marrow R2 values (Fig 1B). Although the mean liver R2 values were similar in the three diseases, it should be noted that half of the patients with SCD had liver R2 values within that of the control group, producing a large standard deviation of R2 values for this organ. The differences in liver R2 values within the SCD data are related to the number of transfusions (see discussion below).

**Table 1:** Proton transverse relaxation rates (R2) of the different tissues from each group of subjects.

	Liver R2(s^-1^)	Bone Marrow R2(s^-1^)	Spleen R2(s^-1^)	Kidney R2(s^-1^)	Heart R2*(s^-1^)
**Control**	33.7 ± 7.6	21.8 ± 4.9	19.6 ± 2.8	17.1 ± 2.9	
	(21–45)	(14.5–34.4)	(14.2–23.5)	(12.2–22)	
**SCD**	123.7 ± 108.1	43.2 ± 21.1	67.2 ± 31.5	27.1 ± 8.7	31.6 ± 6.3
	(26.7–366)	(18.3–105.7)	(23.9–149)	(12.6–54.4)	(22–46.2)
**PNH**	144.3 ± 79.8	36.1 ± 19.2	52.3 ± 31.8	18.1 ± 3.5	33.2 ± 10.6
	(48.8–354.9)	(17.8–90.6)	(20.5–130.9)	(14.6–21.5)	(18–59.8)
**NTDT**	94.7 ± 36.3	32.6 ± 7.0	26.4 ± 8.2	16.1 ± 2.6	26.6 ± 5.2
	(52.5–163.0)	(23.3–45.1)	(11.0–36.3)	(12.6–20.3)	(22.0–33.4)

Numbers correspond to mean ± SD, and those in brackets are the range of values. Statistical differences between the groups are presented in Fig 1.

A more varied pattern of iron accumulation between the different groups of patients is observed in the spleens and kidneys. The mean spleen R2 value in the control group was significantly lower than that in the SCD and PNH patients (Fig 1C). The patients with NTDT did not seem to accumulate as much iron in the spleen relative to the liver and bone marrow when compared with the patients with PNH and SCD. The mean spleen R2 value for the NTDT patients is significantly different from that for the SCD patients (Fig 1C). Significant differences in the mean kidney R2 values were only observed between the patients with SCD and the control group (Fig 1D); mean kidney R2 values were also significantly different between the SCD and NTDT patients (Fig 1D). No cardiac R2* data were available for the control subjects and no significant differences in mean cardiac R2* values were observed between any of the patient groups (Fig 1E).

### Comparison of R2 ratios

**Fig 2:**
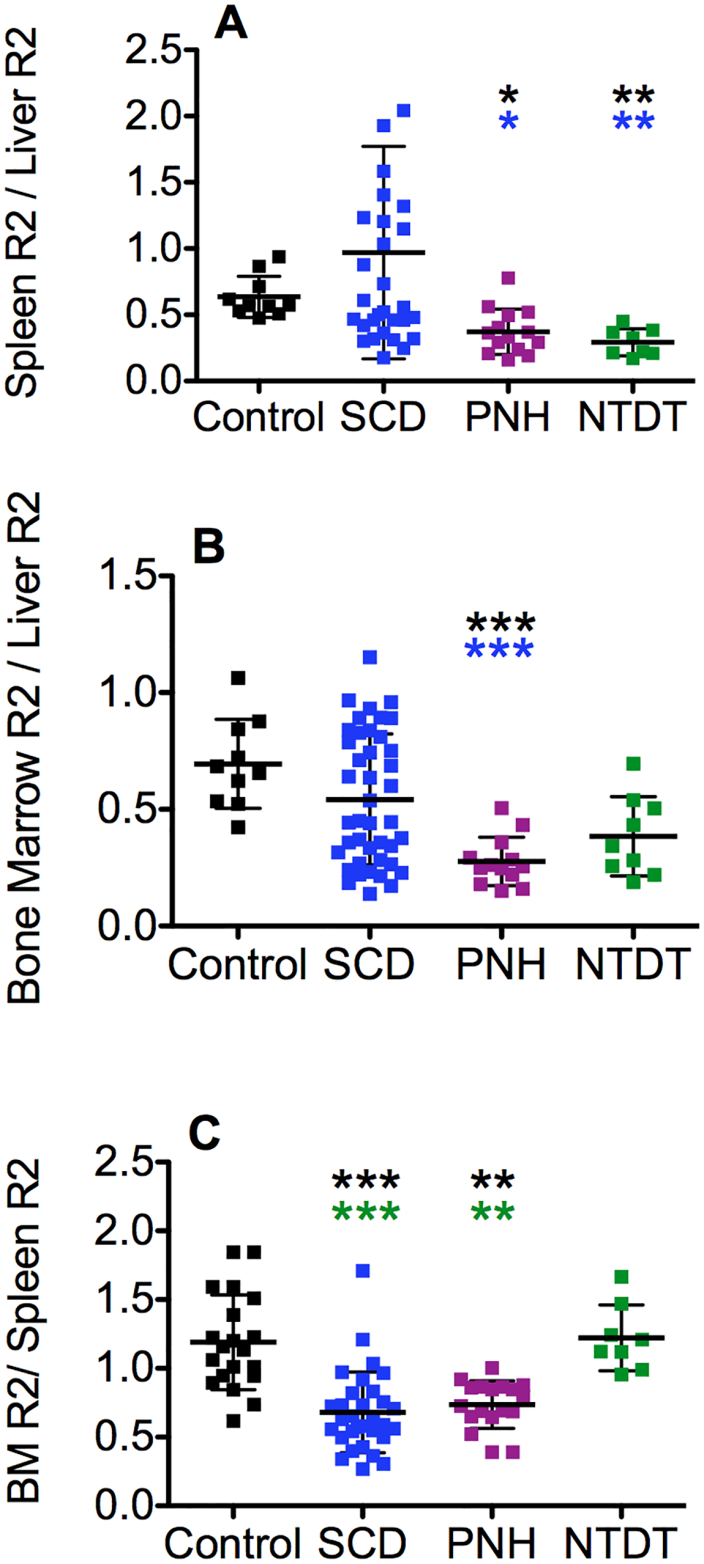
Comparison of tissue R2 ratios for four different groups of subjects: controls (black) and SCD (blue), PNH (purple) and NTDT (green) patients. (A) Spleen R2 / Liver R2, (B) Bone Marrow R2 / Liver R2 and (C) Bone Marrow R2 / Spleen R2. Mean values ± SD are represented by the horizontal bars. (*) p < 0.05, (**) p < 0.01, (***) p < 0.001 for Kruskal Wallis analysis with Dunn post test. The asterisk color indicates the group with which the difference was found. The whole SCD data set has been used for the analysis.

To characterize the biodistribution of the iron loading among the different organs, we evaluated the ratios of R2 in pairs of tissue types for the three groups of patients and control subjects (Table 2 and Fig 2). Ratios were calculated for tissue showing preferential iron accumulation: Spleen R2/ Liver R2, Bone Marrow R2/Liver R2, and Bone Marrow R2 /Spleen R2.

**Table 2:** Mean values and standard deviations of the different tissue ratios for each group of patients.

	Spleen R2 / Liver R2	BM R2/ Liver R2	BM R2 /Spleen R2
**Control**	0.6 ± 0.2	0.7 ± 0.2	1.1 ± 0.3 *(l*, *h)*
**SCD**	0.9 ± 0.8	0.5 ± 0.3	0.7 ± 0.3
***SCD Transfused < 20 units***	*1*.*2 ± 0*.*8*	*0*.*7 ± 0*.*2 (h)*	*0*.*5 ± 0*.*2*
***SCD Transfused > 20 units***	*0*.*8 ± 0*.*4*	*0*.*4 ± 0*.*3 (l)*	*0*.*8 ± 0*.*3*
**PNH**	0.4 ± 0.2 *(l)*	0.3 ± 0.1 *(l)*	0.7 ± 0.3
**NTDT**	0.3 ± 0.1 *(l)*	0.4 ± 0.2	1.2 ± 0.2 *(l*, *h)*

Numbers correspond to mean ± SD. Two subgroups from the SCD patients have been analyzed splitting them into those who had received more or less than 20 top up transfusions. Statistical differences between the whole groups are presented in Fig 2. In this table, only the significant differences found for the low (l, p < 0.05) and high (h, p < 0.05) transfusion SCD sub groups for Kruskal Wallis analysis with Dunn post test are presented.

The tissue R2 ratios from the 4 groups of patients are shown in Fig 2 and Table 2. Regarding the Spleen R2/ Liver R2 ratio, no significant differences have been found between the controls and the SCD patients, when analyzing the whole SCD data set together (Fig 2A). When considering two subgroups within the SCD patients, depending on the number of transfusions received (more or less than 20 top up units) (Table 2), it can be observed that those patients with higher number of transfusions accumulate more iron in the liver relative to spleen in comparison with those patients that had received less transfusions. Comparing the low and high transfused SCD patients with the rest of the groups, the low transfused SCD patients had a spleen/liver R2 ratio that exceeded 1 and that ratio was significantly higher than the PNH and NTDT groups. These differences were not found with the highly transfused SCD patients (Table 2).

Fig 2B and Table 2 show the Bone Marrow R2/Liver R2 ratios. No significant differences have been found between the controls and the whole set of SCD patients but significantly lower Bone Marrow R2/Liver R2 ratios are observed in the PNH patients in comparison with the controls and SCD patients (Fig 2B). These differences remain for the low transfused SCD patients (Table 2), but are not significantly different to the highly transfused ones.

Although, when analyzing each tissue independently, Bone Marrow R2 and Spleen R2 values were higher in the NTDT patients in comparison with the controls (Fig 1), the Bone Marrow R2/Spleen R2 ratios from these two groups are not significantly different (Fig 2C and Table 2). In addition, NTDT patients and controls have significantly higher Bone Marrow R2/Spleen R2 ratios than the SCD (both high and low transfused) and PNH patients (Fig 2C and Table 2).

### Correlations between R2 in different tissues within each group of patients

**Fig 3:**
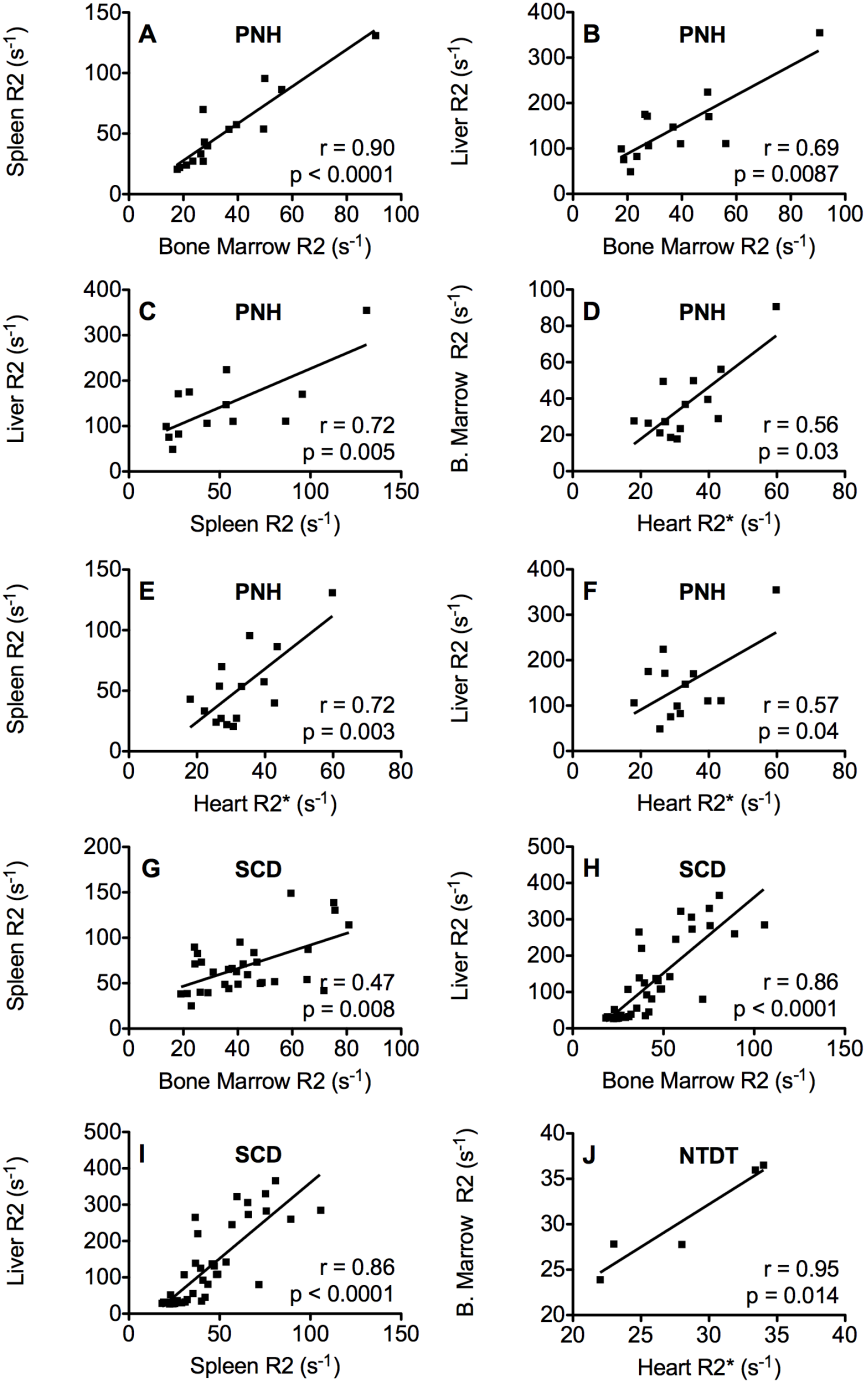
Correlations between MRI R2 values in the different organs in PNH (A-F), SCD (G-I) and NTDT (J) patients.

We analyzed correlations between R2 values of the different tissues for the four groups studied: control subjects, PNH, SCD and NTDT patients (Fig 3). No significant correlations between the different R2 values were found in the control group.

In PNH patients, significant correlations were found between spleen R2 and bone marrow R2 (Fig 3A), liver R2 and bone marrow R2 (Fig 3B), liver R2 and spleen R2 (Fig 3C) bone marrow R2 and heart R2* (Fig 3D), spleen R2 and heart R2* (Fig 3E) and liver R2 and heart R2* (Fig 3F).

In SCD, significant correlations were found between spleen R2 and bone marrow R2 (Fig 3G), liver R2 and bone marrow R2 (Fig 3H), and between liver R2 and spleen R2 (Fig 3I). In these patients, kidney R2 was available, but no correlations were found between kidney R2 and heart R2* or R2 values of the other organs characterized.

In the NTDT patients, a significant correlation was only found between bone marrow R2 and heart R2* (Fig 3J). No significant correlations in iron load were found between tissues of the other organs.

### Correlations of tissue R2 with serum ferritin and iron scores

**Fig 4:**
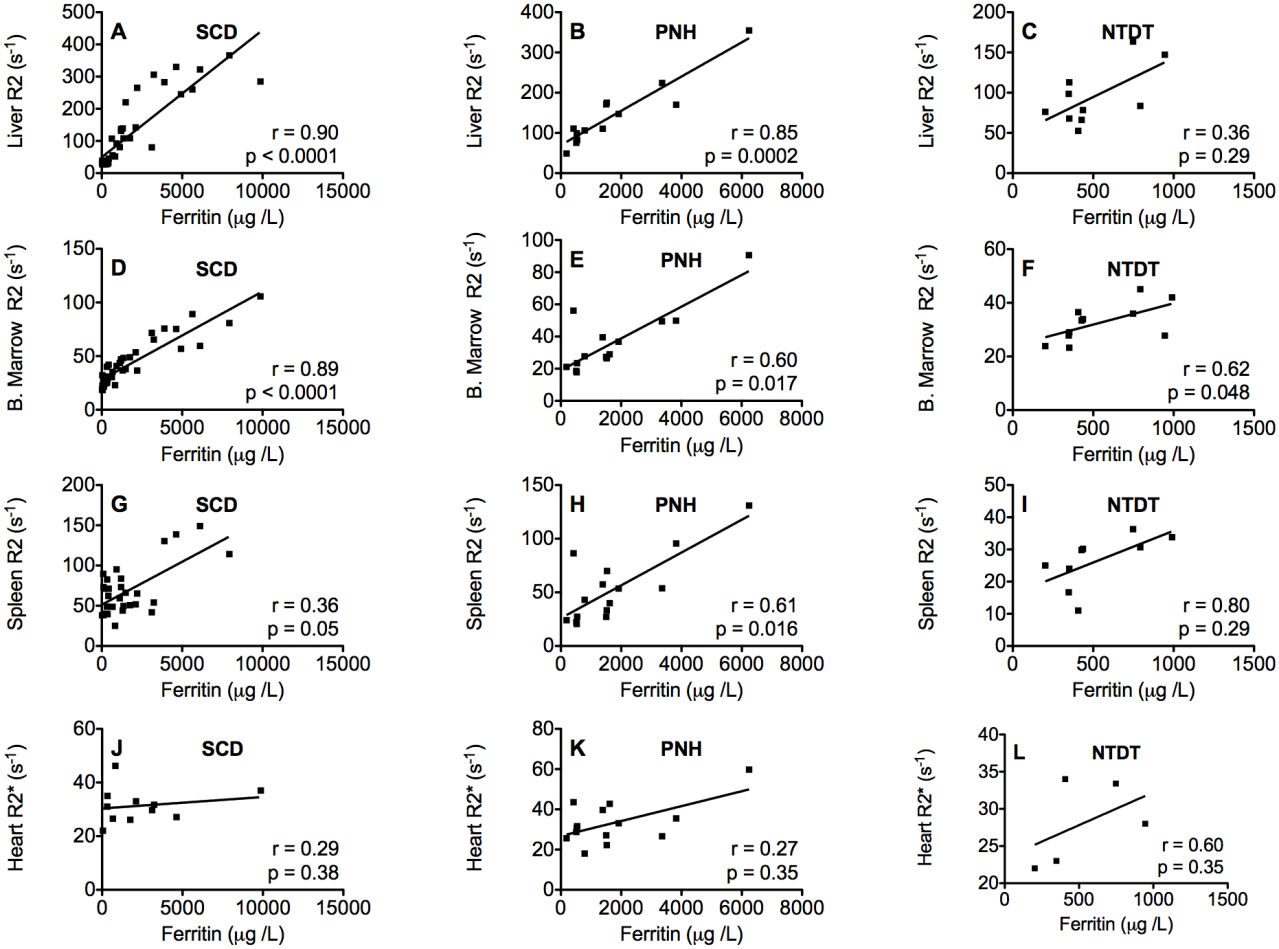
Correlations between serum ferritin concentration and liver R2 (A-C), bone marrow R2 (D-F), spleen R2 (G-I) and heart R2* (J-L) in SCD, PNH and NTDT patients.

The correlations between serum ferritin and the tissue R2 are shown in Fig 4. In patients with SCD, there were no significant correlations between serum ferritin and spleen R2, kidney R2 (not shown) and heart R2*, but significant correlations were found between serum ferritin and liver R2 (p < 0.0001) and bone marrow R2 (p < 0.0001).

In the PNH patients, significant correlations were found between serum ferritin and all the tissues analyzed (liver R2 (p = 0.0002), bone marrow R2 (p = 0.017) and spleen R2 (p = 0.016)) except for heart R2*.

In the NTDT group significant correlations were only found between serum ferritin and bone marrow R2 (p = 0.045).

**Fig 5:**
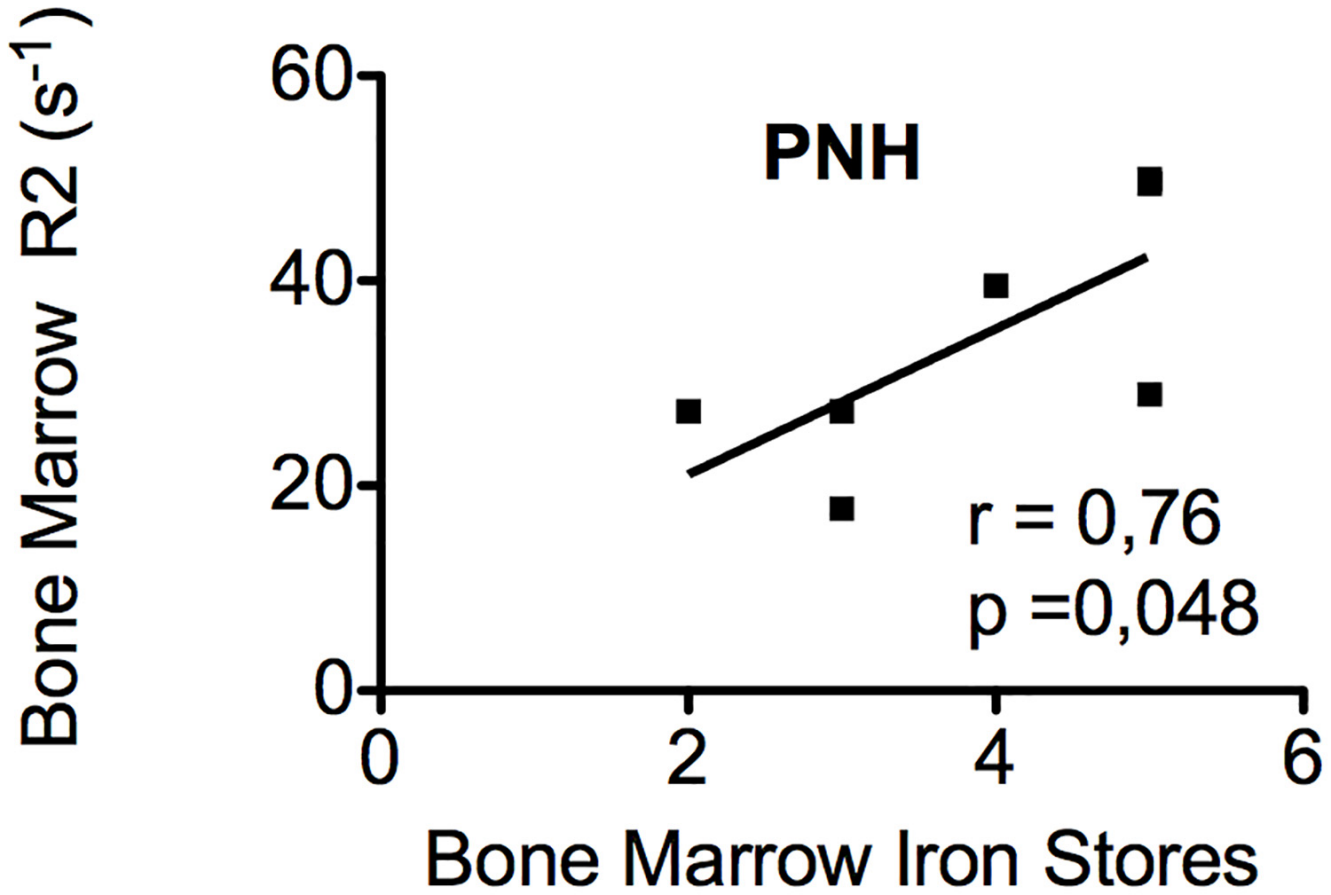
Correlations between bone marrow R2 and bone marrow iron stores determined from bone marrow aspirates (graded in a scale from 1 to 6). Two of the data points of grade 5 superimpose.

Iron scores in bone marrow were available from 7 PNH patients. A weak but still significant correlation was found between bone marrow R2 and bone marrow iron scores from marrow aspirates (p = 0.048) (Fig 5).

## Discussion

### Bone marrow iron accumulation

**Fig 6:**
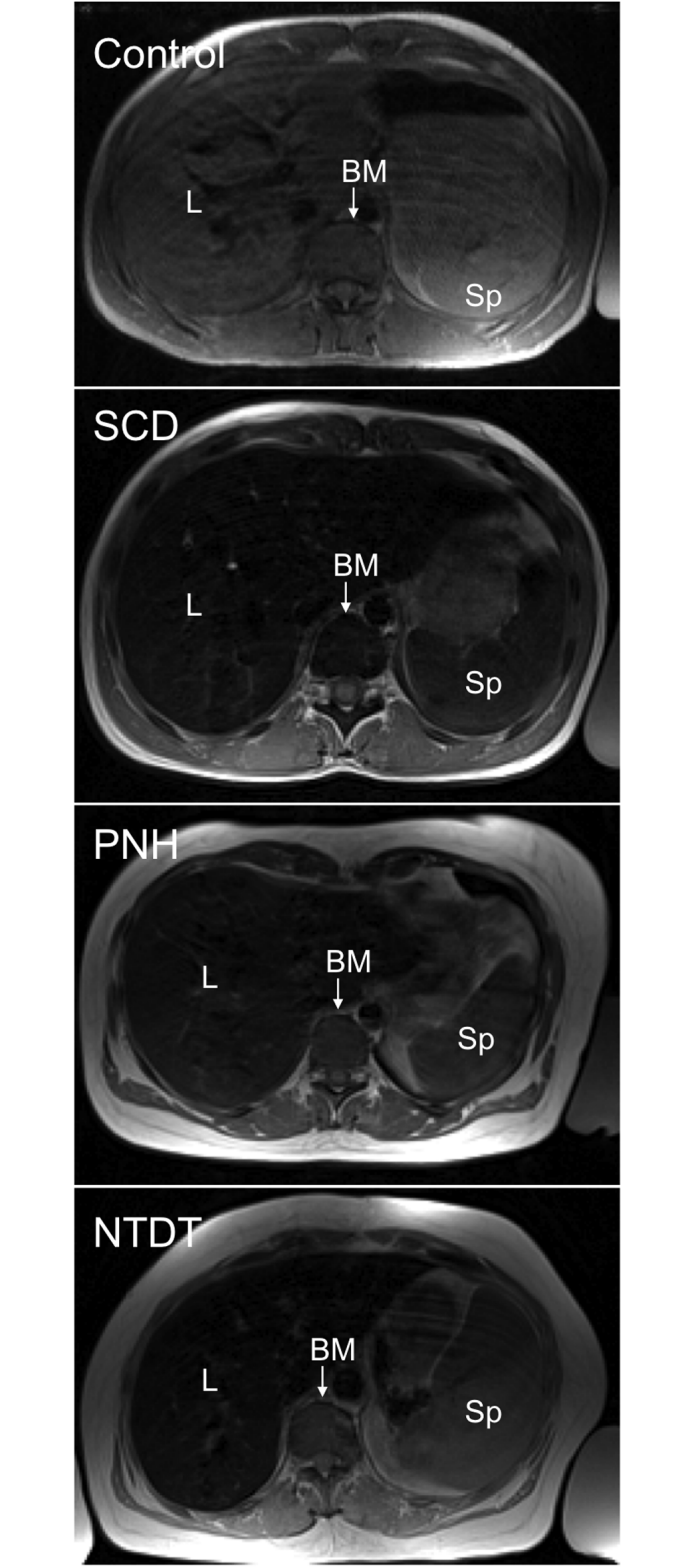
Axial slices of the longest echo time (TE = 18 ms) for subjects from the four different groups characterized. (A) Control, (B) SCD, (C) PNH and (D) NTDT.

We have been able to quantitatively assess a surrogate biomarker of iron accumulation in several organs simultaneously, including the not so frequently characterized bone marrow. Our study of bone marrow R2 in SCD, PNH and NTDT patients has shown increased R2 values for the three groups of patients in comparison with the controls (mean bone marrow R2 values ≈ 1.5–2 fold higher). Even from a careful visual inspection of the MRI images, hypointense bone marrow was detected in many patients (Fig 6). Previous studies on bone marrow R2 in the context of these diseases have confirmed image hypointensity related to iron deposition [23–27], in particular, as a reflection of transfusion therapies that lead to iron accumulation over the years.

In the PNH, SCD and NTDT patients there was generally a good correlation between bone marrow R2 and serum ferritin suggesting that serum ferritin may be a reasonable surrogate marker for bone marrow iron in these diseases. In addition, a significant correlation was found between bone marrow R2 and iron scores in the PNH patients (Fig 5). Still, further studies are needed, on more patients and with different diseases, to validate this non-invasive technique as a surrogate measurement of iron accumulation in bone marrow.

Non-invasive measurements of bone marrow R2 could, nevertheless, add additional confirmation of bone marrow iron status in research or clinical studies. In particular, evaluation of bone marrow iron by R2 measurement could be of benefit before bone marrow transplantation, an emerging curative treatment option for patients with SCD [28, 29]. Recently it has been shown that bone marrow iron load is a risk factor for invasive aspergillosis, a major cause of death after hematopoietic stem cell transplantation [30]. In this context, non-invasive determination of bone marrow iron by MRI may play a key role in confirming serum ferritin observations in patients that require marrow transplants. Abdominal MRI images collected routinely for liver iron concentration measurements by R2 may be used to characterize bone marrow iron accumulation in future clinical practice.

In addition, this characterization protocol will be a useful tool for further studies on the kinetics of iron loading and removal in the different diseases, especially to evaluate different chelating therapies. These measurements will also be of great relevance to evaluate the pattern of iron accumulation in other diseases, providing a more direct measurement of each tissue iron loading than the serum ferritin.

### Serum ferritin as a marker of tissue iron overload

In addition to the good correlation between bone marrow R2 and serum ferritin, moderate to good correlations were found between serum ferritin and the degree of iron loading (as assessed by tissue R2) in the liver for SCD and PNH patients, but not for NTDT ones. Similar to our findings, Papakonstantinou et al. [27] also observed significant correlations between serum ferritin and bone marrow R2 and liver R2 in thalassemia major patients, but Drakonaki et al. [24] did not.

However, a significant correlation between serum ferritin and tissue iron loading does not necessarily indicate that serum ferritin is an accurate predictor of tissue iron concentration (see e.g. [31]). Poor sensitivity and specificity are often encountered even with relatively strong significant correlations. For example, although Brittenham et al. found a significant correlation between plasma ferritin and liver iron concentration in 111 transfused patients with thalassemia and SCD (R = 0.76, p <0.0001), the 95% prediction intervals for liver iron concentration were so broad, such that a single determination of plasma ferritin was unreliable in predicting liver iron concentration.

### PNH iron biodistribution

In PNH, the Bone Marrow R2/ Liver R2 ratio analysis indicates that, for a given level of total body iron stores, the patients accumulate less iron in the bone marrow when compared with controls (Fig 2). This fact is interesting given the significantly higher bone marrow R2 values observed in comparison with the control group (Fig 1). Taken together with the Spleen R2/ Liver R2 ratio, which is also low, we could conclude that the organ that preferentially accumulates iron in the PNH group is the liver. The Bone marrow R2/ Spleen R2 ratio is significantly lower than the controls and NTDT groups, but similar to the SCD patients. This result could imply an imbalance in normal iron distribution that favors the spleen, in preference to the bone marrow, as a result of hemolysis in our PNH cases.

### SCD iron biodistribution

Patients with SCD are the only group in which increased iron accumulation is observed in all 4 organs (liver, bone marrow, spleen and kidneys) in comparison with controls. A wide range of tissue R2 values for this group of patients, however, is observed. We have previously shown that in SCD patients liver iron accumulation is related to transfusion rate while kidney iron accumulation is related to severity of hemolysis [16]. Due to the large range of the tissue R2 data, we have analyzed the impact of the number of transfusions on the biodistribution of the iron accumulation as observed by the tissue R2 ratios (Fig 2 and Table 2).

The mean Bone Marrow R2/Liver R2 ratio from SCD patients that received a low number of transfusions was similar to the control group and significantly higher than the PNH patients. Conversely, the mean Bone Marrow R2/Spleen R2 ratio from SCD patients that received a low number of transfusions was significantly lower than the control and NTDT groups, but similar to the PNH patients. Interpretation of the Spleen R2/Liver R2 ratio in the SCD patients is complicated by the large spread in values, even when the patients are split into low and high transfusion groups. However, in the low transfused SCD group the mean Spleen R2/Liver R2 ratio is significantly higher than in the PNH and NTDT groups and double the control mean value without reaching statistical significance.

Together, these high Spleen R2/Liver ratios, normal Bone Marrow R2/Liver R2 ratios, and low Bone Marrow R2/Spleen R2 ratios suggest preferential iron loading of the spleen in the low transfusion SCD group. However, when the number of transfusions increases, the SCD biodistribution shifts towards the one observed for the PNH patients, suggesting a greater iron accumulation in the liver i.e. a parenchymal loading. Brewer et al. speculated that spleen iron loading may be related to the enhanced trapping of the rigid sickle cells in spleen vascular and sinusoidal spaces, which would be consistent with predominant loading in the reticuloendothelial cells in spleen [32].

It has been suggested that the significantly higher mean kidney R2 observed for the SCD patients is explained by the ongoing intravascular hemolysis, releasing cell-free hemoglobin into the circulation. The cell-free plasma hemoglobin may then bind to haptoglobin forming complexes that are absorbed by the reticuloendothelial system [33]. Free heme may also be complexed by hemopexin [34] and transported mainly to the liver. In situations of ongoing chronic hemolysis (e.g. SCD), when the binding capacity of haptoglobin and hemopexin is saturated, free heme and free hemoglobin are delivered to the kidney [34, 35]. Kidney iron accumulation associated with hemolysis has also been observed in PNH patients using invasive biopsy [36] and non-invasive qualitative MRI [37]. Unfortunately, not enough MR images from the kidneys could be obtained from the scans of our PNH group of patients for their subsequent analysis.

### NTDT iron biodistribution

We have found a reduced iron accumulation in the spleen of the NTDT patients, similar to the control group, as opposed to the other two diseases. The spleen is usually enlarged to varying degrees in patients with β-thalassemia intermedia, but not iron-loaded [38]. Iron accumulation in β-thalassemia intermedia occurs mainly from increased intestinal iron absorption triggered by the chronic anemia and ineffective erythropoiesis [39, 40], which we observed as increased mean R2 in the liver and bone marrow.

The Spleen R2/Liver R2 ratio analysis, supports the low Spleen R2 values, indicating a reduced iron accumulation in the spleen in comparison with the liver iron levels. Interestingly, the Spleen R2/ Liver R2 ratios in NTDT are not significantly different from those in PNH.

The similar Bone Marrow R2/Spleen R2 ratio found for the NTDT and control groups (Fig 2C) may reflect an iron loading mainly from natural routes like gut absorption for the NTDT group. When the iron loading is a consequence of hemolysis as in the low transfused SCD patients, iron accumulates relatively more in macrophages in the spleen, leading to significantly lower Bone Marrow R2/Spleen R2 ratio values for the these patients in comparison with the NTDT and controls (Fig 2C).

Finally, it should be mentioned that although MRI can estimate total iron accumulation in each organ, it cannot discriminate iron accumulation within different cells from a given organ.

## Conclusions

We have used non-invasive MRI to quantify a surrogate biomarker of iron accumulation in liver, spleen, bone marrow and kidneys simultaneously. Of special interest is the quantitative analysis of bone marrow, which is not so frequently characterized by this technique.

Our study of liver and bone marrow R2 in SCD, PNH and NTDT patients has shown increased R2 values for the three groups of patients in comparison with the controls. Only spleen R2 values from SCD and PNH patients were significantly different from the controls. The pattern of iron accumulation in the different tissues is strongly related to the source of iron accumulation associated with each disease. The PNH group preferentially accumulates iron in the liver secondary to blood transfusions. Preferential liver iron loading is also found in the highly transfused SCD patients, while the low transfused ones present a preferential iron loading of the spleen, consistent with macrophage scavenging. For NTDT patients, a reduced spleen iron accumulation in comparison with the liver and bone marrow loading has been found, related to the increased intestinal iron absorption.

Although moderate to good correlations have been found between the degree of iron loading (as assessed by tissue R2) for the liver and bone marrow and serum ferritin, this does not necessarily indicate that serum ferritin is an accurate predictor of tissue iron concentration. Tissue R2 measurements are a more direct measurement of each tissue iron loading, being useful to evaluate the pathways of iron flow between the different organs.
